# Generalized gingivitis-related salivary proteomic profile in pregnant women with obesity: insights into biological mechanisms assessed by Tandem Mass Spectrometry

**DOI:** 10.1590/1678-7757-2025-0031

**Published:** 2025-05-12

**Authors:** Laura Teodoro de MARCHI, Amanda Borges PIRONDI, Talita Mendes Oliveira VENTURA, Larissa Tercilia GRIZZO, Marília Afonso Rabelo BUZALAF, Gerson Aparecido FORATORI-JUNIOR

**Affiliations:** 1 Universidade de São Paulo Faculdade de Odontologia de Bauru Departamento de Ciências Biológicas Bauru São Paulo Brasil Universidade de São Paulo, Faculdade de Odontologia de Bauru, Departamento de Ciências Biológicas, Bauru, São Paulo, Brasil

**Keywords:** Gingivitis, Obesity, Pregnancy, Proteomics, Saliva

## Abstract

**Aim:**

This cross-sectional study investigated the salivary proteomic profile associated with generalized gingivitis in pregnant women with obesity.

**Methodology:**

Pregnant women in the third trimester (≥27 weeks of gestation) were divided into two groups based on bleeding on probing (BOP): G1 (BOP>50%; n=9) and G2 (BOP 0–30%; n=9). Collected unstimulated saliva samples were individually analyzed using nano liquid chromatography electron spray ionization tandem mass spectrometry. Identified proteins were classified according to gene ontology for biological processes, molecular functions, immune system involvement, and cellular components. Differential protein expression was determined using thresholds of *p*<0.05 for downregulation and 1-*p*>0.95 for up-regulation proteins.

**Results:**

Of the 183 identified proteins, 100 were shared between groups, totaling 57 up-regulated and 27 downregulated proteins in G1. Key biological processes included antimicrobial humoral response and hydrogen peroxide catabolism, with proteins linked to immune function and endopeptidase regulation. Functional analysis showed that *Lactotransferrin* (5-fold increase in G1), *Haptoglobin* (4-fold), and *Immunoglobulin J chain* (3-fold) were up-regulated, whereas *Statherin* (5-fold) and *Protein S100-A8* (4-fold) were downregulated in G1.

**Conclusions:**

Pregnant women with obesity and generalized gingivitis exhibited a distinct salivary proteomic profile characterized by the up-regulation of immune-related proteins and downregulation of tissue-protective proteins. These findings suggest potential salivary biomarkers for detection and targeted management of periodontal inflammation in this high-risk population.

## Graphical abstract

**Figure:**
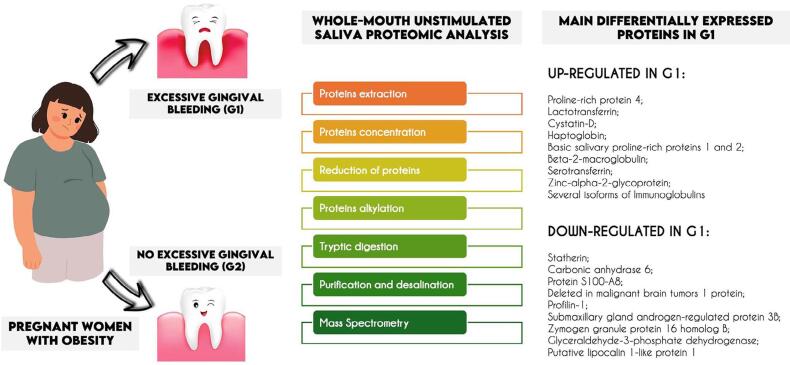

## Introduction

Pregnancy induces significant hormonal changes, particularly elevated progesterone and estrogen levels which, in the presence of dental biofilm, exacerbate inflammation in periodontal tissues. This hormonal environment suppresses the immune response, increasing the risk of periodontal diseases in pregnant women.^1^ Gingivitis is the most common periodontal condition during pregnancy, with a prevalence ranging from 60% to 75%.^2^ Its progression is often observed from the second trimester onward, driven by hormonal fluctuations and reduced antimicrobial activity of neutrophils, which weakens the immune system.^3^ Pregnancy alone does not cause gingivitis, as this is a biofilm-dependent oral disease; however, the immunological changes associated with this period make women more susceptible to an exacerbated local inflammatory response, even in the presence of a reduced amount of dental biofilm.^4^

A growing global health concern, obesity exacerbates the risk of periodontal diseases by inducing a systemic inflammatory state via production of pro-inflammatory cytokines, adipokines, and other bioactive molecules by adipose tissue. Additionally, obesity alters immune cell function, including T lymphocytes and macrophages, impairing the body’s ability to control oral inflammation, even in the presence of minimal dental biofilm.^5^^-^^6^ Consequently, pregnant women with obesity may experience more severe gingival inflammation due to the combined effects of hormonal, immunological, and metabolic dysregulation.

Despite the recognized impact of these factors on periodontal health during pregnancy, most studies have focused on periodontitis and its systemic implications like gestational diabetes mellitus, arterial hypertension and preterm birth.^7^^-^^12^ Biological mechanisms analyzed through saliva associating gingivitis and pregnancy remain poorly understood, particularly in the context of obesity. Proteomic analysis has emerged as a powerful tool to elucidate such mechanisms, offering insights into protein expression, interactions, and functions within biological systems. This approach has been instrumental in identifying biomarkers for various diseases and is particularly suited for non-invasive diagnostic fluid analysis, such as saliva.

Proteomic findings have shown that pregnancy-associated gingivitis involves heightened neutrophil-mediated immune responses and compromised antioxidant defenses, with reduced salivary cystatin C levels predisposing pregnant women to gingival inflammation.^13^ However, these analyses have not yet been extended to pregnant women with obesity, a group that may present distinct proteomic profiles due to the interplay between systemic inflammation and local oral conditions.

To the best of our knowledge, no previous studies have used advanced Tandem Mass Spectrometry to investigate the salivary proteomic profile associated with gingivitis in pregnant women with obesity. This study seeks to fill this gap by identifying salivary proteins linked to generalized gingival bleeding in this population, providing new insights into the underlying biological mechanisms and potential biomarkers for improved diagnosis and management.

## Methodology

This cross-sectional study followed the Strengthening the Reporting of Observational Studies in Epidemiology (STROBE) guidelines^14^ and was registered on ReBEC (https://ensaiosclinicos.gov.br/rg/RBR-9mtvmqj) (registered on 13 August 2024) under protocol number RBR-9mtvmqj and Universal Trial Number (UTN) U1111-1309-1168.

### Ethical approval

It was approved by the Internal Research Ethics Committee (CAAE 77660724.3.0000.5417; protocol code 6.705.259; approval on March 6, 2024) according to protocol established by the Declaration of Helsinki published in 1975 and revised in 2013. Individuals were included only after approval and signature of the written informed consent form.

### Sample selection

A total of 18 pregnant women in their third trimester (27^th^-39^th^ gestational week) were consecutively recruited from March to September 2024 at Primary Healthcare units in Bauru, São Paulo, Brazil. Eligibility criteria included: pregnant women with pre-pregnancy obesity (BMI ≥ 30 kg/m^2^) aged 18-40 years, regular prenatal care, adequate cognitive function, and no condition requiring bed rest. Exclusion criteria comprised: twin pregnancy, hypertensive disorders (blood pressure ≥140/90 mmHg) or preeclampsia, gestational diabetes mellitus (fasting glucose ≥92 mg/dL; ≥180 mg/dL at 1 hour; ≥153 mg/dL at 2 hours), malnutrition (BMI<18.5 kg/m^2^), overweight but not obese (BMI 25.0-29.9 kg/m^2^), suspected or confirmed SARS-CoV-2 infection, reduced salivary flow (<0.25 mL/min), smoking or alcohol use during pregnancy, medications impacting periodontal health or salivary flow, ongoing orthodontic, periodontal, or other dental treatments, diagnosed periodontitis (defined as detectable interdental attachment loss in ≥2 non-adjacent teeth, or buccal or oral attachment loss of ≥3 mm with a periodontal pocket >3 mm in ≥2 teeth, not attributable to non-periodontal causes^15^), and multiple tooth loss (more than two teeth per hemiarch). Patients with a history of periodontal treatment who were classified as supportive periodontal patients (reduced periodontium indicative of attachment loss due to recession) were excluded from this study. Conversely, individuals with gingival recession unrelated to periodontal disease (e.g., trauma-induced recession) were included since this condition would not introduce bias into the study outcomes.

Based on previous studies,^7^^-^^10^ the participants should have pre-pregnancy BMI equal to or higher than 30.0 kg/m^2^ which was obtained from pre-natal medical files. Afterwards, the women were allocated into groups according to gingival bleeding prevalence assessed in a dichotomous manner (positive or negative) based on the percentage of sites with bleeding on probing (BOP) following a full-mouth periodontal examination, considering six sites per tooth (three on the buccal and three on the palatal/lingual surfaces). This approach aligned with validated bleeding index,^16^ ensuring reliability and reproducibility in gingival inflammation assessment.

According to Trombelli, et al.^17^ (2018), BOP in ≥30% of sites characterizes generalized gingivitis. However, patients at the lower threshold (e.g., 31% BOP) likely exhibit a salivary proteomic profile similar to those with 29% BOP, despite being classified into different groups. Consequently, including participants with 30–50% BOP could obscure biomolecular distinctions associated with disease progression. To ensure a clearer identification of biological processes related to more severe gingival inflammation, only patients with BOP ≥50% were included in the study although those with 30–50% BOP also met the clinical criteria for generalized gingivitis.

But despite excluding this range, we adhered to the standardized gingivitis classification proposed by Trombelli, et al.^17^ (2018), and this modification was made solely to prevent potential misinterpretation of biomolecular outcomes. Accordingly, pregnant women were classified as having generalized gingivitis if BOP in ≥50% of gingival sites (G1=9), whereas those without generalized gingivitis presented BOP in 0-30% of sites (G2=9).

### Co-variables

Co-variables were analyzed to ensure sample homogeneity, minimizing potential biases in the proteomic profile analysis. Data on age, schooling level,^12^ household monthly income,^12^ oral hygiene behaviors (daily tooth brushing and flossing), prevalence of dental surfaces with biofilm, probing pocket depth (PPD), and clinical attachment level (CAL) were collected. One calibrated dentist performed the full-mouth periodontal examination and saliva collection. Calibration involved examining 15 individuals, categorizing them as with or without periodontitis and with or without gingivitis (κ=0.95). Intra-examiner reliability for probing depth measurements was also assessed using the Intraclass Correlation Coefficient (ICC=0.88), with examinations repeated after a 15-day interval to minimize potential alterations in periodontal fibers following initial probing.

### Saliva collection

Unstimulated whole-mouth saliva was collected before the periodontal examination. To account for circadian rhythm, collections took place in the morning (09:00-11:00). Participants were instructed to refrain from eating or drinking before the appointment and to clean their mouths thoroughly before saliva collection. After rinsing with 5 mL of deionized water and expelling it, they passively drooled into a sterilized 50 mL plastic Falcon tube kept on ice for 10 minutes to measure the unstimulated salivary flow.^18^^-^^21^ Immediately after collection, saliva was centrifuged at 4500 x *g* for 15 minutes at 4°C to remove debris. The supernatant from each sample was then stored at -80ºC until analysis.^18^^-^^21^

### Sample preparation for proteomic analysis

Proteomic analysis was conducted based on a previous protocol.^18^ Saliva samples were analyzed individually, and the proteins from each sample (1000 µL each) were extracted using 1000 µL of an extraction solution containing 6M urea, 2M thiourea in 50 mM NH_4_HCO_3_, pH 7.8. Samples were vortexed for 10 minutes at 4ºC, sonicated for 5 minutes, and centrifuged at 20,817 x *g* at 4ºC for 10 minutes. This 3-step cycle was repeated three times. Next, samples were concentrated in Amicon tubes (Amicon Ultra-15 Centrifugal Filter Units – Merck Millipore^®^, Tullagreen, County Cork, Ireland) to a volume of approximately 150 μL. A 1 μL aliquot of each sample was taken to quantify total protein using the Bradford method (Bio-Rad, Hercules, California, United States). Proteins were reduced with 5 mM dithiothreitol for 40 minutes at 37°C and alkylated with 10 mM iodoacetamide for 30 minutes in the dark. Samples were digested for 14 hours at 37°C by adding 2% (w/w) trypsin (Thermo Scientific Pierce Trypsin Protease, Rockford, Illinois, United States). Digestion was stopped by adding 10 μL of 5% trifluoroacetic acid; afterwards the samples were desalted and purified using C18 Spin Columns (Thermo Scientific, Rockford, Illinois, United States). Samples were then resuspended in a solution containing 3% acetonitrile and 0.1% formic acid and subjected to mass spectrometry (nanoLC-ESI-MS/MS) (Waters, Manchester, New Hampshire, United Kingdom).

### Shotgun label-free quantitative proteomic analysis (nLC-ESI-MS/MS)

Peptide identification was performed using a Xevo G2 QTof mass spectrometer coupled with a nanoACQUITY system (Waters Co., Manchester, New Hampshire, United Kingdom), operating in positive ion nanoelectrospray mode. Data were collected by the MSE high-energy method (19-45 V), enabling simultaneous acquisition of both precursor and fragment ions in a single injection. Data acquisition scan range was 50–2000 Da. A [Glu1] fibrinopeptide solution (1 pmol/µL) at 0.5 µL/min flow rate served as the lock spray for accuracy and reproducibility.^18^

ProteinLynx GlobalServer (PLGS) version 3.0.3 (Waters Co., Manchester, United Kingdom) processed and searched continuous LC-MSE data. Proteins were identified using its ion-counting algorithm which incorporates Monte Carlo simulations and Bayesian probability adjustments to estimate differential expression. A search was performed against the *Homo sapiens* database (reviewed entries only), available on UniProt (Uni-ProtKB/Swiss-Prot, http://www.uniprot.org/ accessed in October 2024), with each protein analyzed by its accession number. Duplicates, reverse sequences, and fragments were excluded. All proteins identified with greater than 95% confidence were included in the quantitative analysis. Expression difference for up-regulated proteins was defined as 1-p>0.95 and for down-regulated proteins as p<0.05.^18^^-^^21^ Differences in expression between groups were analyzed using *t*-test (p<0.05).

### Statistical analysis and Bioinformatics

Sample size calculation, using G*Power 3.1, was based on previous protocol of *in vivo* individual salivary proteomic analyses by mass spectrometry^18^^-^^21^ and on salivary protein abundance from healthy young individuals and those with gingivitis.^22^ These studies were selected due to their methodological similarity to our research^18–21^ and the biological relevance of protein abundance changes in gingival inflammation.^22^ Considering α=0.05 and 1-β=0.8, effect size (difference in total protein between healthy and gingivitis cases) was estimated at 1.4, resulting in a required sample size of 8 participants per group. To account for potential sample losses due to laboratory processing issues, we increased the sample size by 10%, totaling 9 participants per group.

After protein quantification, we performed a post-hoc power analysis based on our data of total protein between groups. With α=0.05 and 1-β=0.8 effect size was 0.95, yielding an achieved power of 62% which would indicate a limited sample size. However, sample size calculation should primarily be based on the primary outcomes. Since this is an exploratory study aimed at identifying multiple differentially expressed proteins in saliva, no single predefined parameter can be used to determine sample size. Estimating effect sizes based on total protein abundance data from previous studies with comparable populations to guide sample size calculation, as we did here, is a usual practice. Nonetheless, when the objective is to identify differentially expressed proteins using untargeted mass spectrometry, total protein abundance should not be considered the primary outcome. Instead, ensuring rigorous methodological criteria during participant recruitment, as detailed in our methodology section, is essential to obtain homogeneous groups and minimize bias.

Statistical analysis was performed using Jamovi (Version 2.6, Computer Software, retrieved from https://www.jamovi.org, accessed in November 2024) and IBM SPSS (Version 25.0, IBM Corp, Armonk, New York, United States). Variables were tested for normality and homogeneity of variance using Shapiro-Wilk and Levene, respectively. *T*-test was used to compare quantitative variables with normality between groups (age, pre-pregnancy and pregnancy BMI, gestational weight gain, daily tooth brushing, dental biofilm-%, BOP-%, salivary flow-mL/min, total protein-ug/ptn). Mann-Whitney was applied to compare quantitative variables without normality and ordinal qualitative variables between groups (schooling level, household monthly income, daily flossing, PPD-mm, CAL-mm).

Gene ontology (GO) was considered to analyze protein categories which, in turn, was based on biological processes, immune system processes, molecular functions, and cell components using ClueGo^®^ plugins of the Cytoscape^®^ 3.8.2 (Institute of Systems Biology, Seattle, Washington, United States). Proteins identified with differential expression (up- and downregulated) in the group comparison had their function distribution analyzed. Significance (κ=0.04) and distribution terms were based on the percentage of the number of associated genes. Mass spectrometric proteomic data was deposited to the ProteomeXchange Consortium via the PRIDE partner repository under data set identifier PXD058171 (approved on 22 November 2024).

STRING^®^ database (https://string-db.org/cgi/network.pl) was accessed for the interaction networks, establishing the interaction network of up- and downregulated proteins (with changes greater or less than 2-fold) in G1 compared with G2.

## Results

We found no significant differences between the groups regarding age, socioeconomic status, anthropometric parameters, oral hygiene behaviors, PPD, CAL, salivary flow, or salivary protein quantification (*p*>0.05), highlighting the effectiveness of the sample recruitment process in achieving high homogeneity and reducing bias in interpreting salivary proteomic data. Most participants had completed tertiary education and reported a monthly income of up to two minimum wages (Table 1).

**Table 1:** Sample contextual and oral characteristics.

	G1 (n = 9)	G2 (n = 9)	p
	Mean ± SD	Mean ± SD	
	Median [1^st^-3^rd^ quartiles]	Median [1^st^-3^rd^ quartiles]	
**Age (years)**	26.80±4.35	26.20±8.15	0.859*
**Schooling level – n (%)**	4 [4 – 5]	4 [3 – 4]	0.256^†^
Illiteracy (0)	0 (0)	0 (0)	
Incomplete primary education (1)	0 (0)	1 (11.11)	
Complete primary education (2)	0 (0)	1 (11.11)	
Incomplete high school (3)	2 (22.22)	1 (11.11)	
Complete high school (4)	4 (44.45)	5 (55.56)	
Incomplete higher education (5)	1 (11.11)	0 (0)	
Complete higher education (6)	0 (0)	1 (11.11)	
Specialization (7)	1 (11.11)	0 (0)	
Master’s degree (8)	0 (0)	0 (0)	
PhD (9)	1 (11.11)	0 (0)	
**Household monthly income**	2 [2 – 3]	2 [1 – 3]	0.680^†^
Up to one MW (1)	1 (11.11)	3 (33.34)	
1–2 MW (2)	5 (55.56)	2 (22.22)	
2–3 MW (3)	1 (11.11)	2 (22.22)	
3–4 MW (4)	0	1 (11.11)	
4–5 MW (5)	0	1 (11.11)	
More than 5 MW (6)	2 (22.22)	0	
**Pre-pregnancy BMI (kg/m**^**2**^**)**	31.10±3.27	34.50±3.23	0.141*
**Pregnancy BMI (kg/m**^**2**^**)**	33.40±4.75	36.30±4.25	0.197*
**Gestational weight gain (kg)**	6.54±5.22	5.97±4.97	0.815*
**Daily toothbrushing**	2.89±0.60	2.56±0.53	0.229*
**Daily flossing**	0 [0 – 1]	1 [0 – 2]	0.534^†^
**Dental biofilm (%)**	57.20±20.90	46.30±23.80	0.316*
**BOP (%)**	56.70±8.38	23.60±6.08	< 0.001*
**PPD (mm)**	2.09 [2.04 – 2.10]	2.02 [1.99 – 2.04]	0.353^†^
**CAL (mm)**	2.10 [2.04 – 2.11]	2.06 [2.02 – 2.07]	0.426^†^
**Salivary flow (mL/min)**	0.54±0.06	0.57±0.04	0.309*
**Total protein (ug/ptn)**	34.10±12.00	46.80±14.70	0.063*

G1, obesity and generalized gingivitis; G2, obesity without generalized gingivitis; SD, standard deviation; P, significance level; MW, minimum wage; BMI, body mass index; BOP, bleeding on probing; PPD, probing pocket depth; CAL, clinical attachment level; * *t* test; † Mann-Whitney.

**Figure 1:**
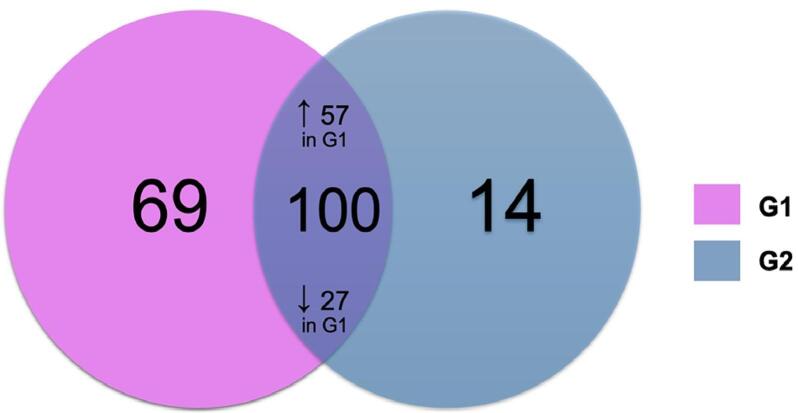
Venn diagram showing common proteins between G1 and G2 samples (including both up- and downregulated proteins in G1), and the number of proteins uniquely identified in each group.

Figure 1 presents a Venn diagram illustrating the total identification of 183 proteins. Among these, 69 were unique to G1 and 14, to G2 (Table S1). Of the 100 proteins shared between the groups, 57 were up-regulated and 27 were downregulated in G1 (Table 2 and 3, respectively).

**Table 2:** Key up-regulated proteins identified in G1 compared with G2.

Accession number	Protein name	Gene	Score	Fold Change	Log(e)	SD	p	ED
**Q16378**	***Proline-rich protein 4***	***PRR4***	**711**	**8.58**	**2.15**	**0.03**	**< 0.01**	↑
**P01861**	***Immunoglobulin heavy constant gamma 4***	***IGHG4***	**24**	**5.75**	**1.75**	**0.06**	**< 0.01**	↑
**P02788**	***Lactotransferrin***	***LTF***	**175**	**4.81**	**1.57**	**0.1**	**< 0.01**	↑
**P28325**	***Cystatin-D***	***CST5***	**1997**	**4.53**	**1.51**	**0.04**	**< 0.01**	↑
**P01859**	***Immunoglobulin heavy constant gamma 2***	***IGHG2***	**24**	**4.14**	**1.42**	**0.14**	**< 0.01**	↑
**P00738**	***Haptoglobin***	***HP***	**78**	**3.94**	**1.37**	**0.06**	**< 0.01**	↑
**P0DOX6**	***Immunoglobulin mu heavy chain***	***IGM***	**119**	**3.56**	**1.27**	**0.06**	**< 0.01**	↑
**P01871**	***Immunoglobulin heavy constant mu***	***IGHM***	**161**	**3.46**	**1.24**	**0.04**	**< 0.01**	↑
**A0M8Q6**	***Immunoglobulin lambda constant 7***	***IGLC7***	**983**	**3.22**	**1.17**	**0.05**	**< 0.01**	↑
**P01591**	***Immunoglobulin J chain***	***JCHAIN***	**2181**	**2.92**	**1.07**	**0.03**	**< 0.01**	↑
**P0DOX7**	***Immunoglobulin kappa light chain***	***IGK***	**1629**	**2.69**	**0.99**	**0.04**	**< 0.01**	↑
**P01834**	***Immunoglobulin kappa constant***	***IGKC***	**1802**	**2.69**	**0.99**	**0.04**	**< 0.01**	↑
**P02812**	***Basic salivary proline-rich protein 2***	***PRB2***	**1467**	**2.53**	**0.93**	**0.02**	**< 0.01**	↑
**P61769**	***Beta-2-microglobulin***	***B2M***	**222**	**2.44**	**0.89**	**0.15**	**< 0.01**	↑
**P69905**	***Hemoglobin subunit alpha***	***HBA1; HBA2***	**335**	**2.39**	**0.87**	**0.1**	**< 0.01**	↑
**P0CG38**	***POTE ankyrin domain family member I***	***POTEI***	**81**	**2.29**	**0.83**	**0.18**	**< 0.01**	↑
**P04280**	***Basic salivary proline-rich protein 1***	***PRB1***	**1130**	**2.25**	**0.81**	**0.06**	**< 0.01**	↑
**P0CF74**	***Immunoglobulin lambda constant 6***	***IGLC6***	**1163**	**2.25**	**0.81**	**0.13**	**< 0.01**	↑
**P02787**	***Serotransferrin***	***TF***	**121**	**2.23**	**0.8**	**0.04**	**< 0.01**	↑
**P25311**	***Zinc-alpha-2-glycoprotein***	***AZGP1***	**138**	**2.23**	**0.8**	**0.22**	**< 0.01**	↑
P02042	*Hemoglobin subunit delta*	*HBD*	293	1.90	0.64	0.16	< 0.01	↑
Q6P5S2	*Protein LEG1 homolog*	*LEG1*	933	1.86	0.62	0.14	< 0.01	↑
P52209	*6-phosphogluconate dehydrogenase, decarboxylating*	*PGD*	79	1.86	0.62	0.16	< 0.01	↑
P68871	*Hemoglobin subunit beta*	*HBB*	554	1.84	0.61	0.1	< 0.01	↑
P01877	*Immunoglobulin heavy constant alpha 2*	*IGHA2*	2097	1.82	0.6	0.01	< 0.01	↑
P01023	*Alpha-2-macroglobulin*	*A2M*	72	1.80	0.59	0.1	< 0.01	↑
P0DOX2	*Immunoglobulin alpha-2 heavy chain*	*IGA2*	1985	1.80	0.59	0.01	< 0.01	↑
P01876	*Immunoglobulin heavy constant alpha 1*	*IGHA1*	4673	1.80	0.59	0.01	< 0.01	↑
P04080	*Cystatin-B*	*CSTB*	1334	1.62	0.48	0.14	< 0.01	↑
P69892	*Hemoglobin subunit gamma-2*	*HBG2*	237	1.60	0.47	0.14	0.02	↑
P69891	*Hemoglobin subunit gamma-1*	*HBG1*	237	1.60	0.47	0.23	0.03	↑
P01833	*Polymeric immunoglobulin receptor*	*PIGR*	1682	1.55	0.44	0.04	< 0.01	↑
P06733	*Alpha-enolase*	*ENO1*	226	1.54	0.43	0.08	< 0.01	↑
P01860	*Immunoglobulin heavy constant gamma 3*	*IGHG3*	86	1.51	0.41	0.07	< 0.01	↑
P02100	*Hemoglobin subunit epsilon*	*HBE1*	237	1.51	0.41	0.18	< 0.01	↑
P01034	*Cystatin-C*	*CST3*	1163	1.49	0.4	0.07	< 0.01	↑
P01857	*Immunoglobulin heavy constant gamma 1*	*IGHG1*	122	1.43	0.36	0.14	< 0.01	↑
P68133	*Actin, alpha skeletal muscle*	*ACTA1*	246	1.39	0.33	0.07	< 0.01	↑
Q8TAX7	*Mucin-7*	*MUC7*	319	1.39	0.33	0.07	< 0.01	↑
P59665	*Neutrophil defensin 1*	*DEFA1; DEFA1B*	514	1.39	0.33	0.15	0.03	↑
Q9BYX7	*Putative beta-actin-like protein 3*	*POTEKP*	132	1.36	0.31	0.13	0.01	↑
P68032	*Actin, alpha cardiac muscle 1*	*ACTC1*	246	1.36	0.31	0.07	< 0.01	↑
Q502W6	*von Willebrand factor A domain-containing protein 3B*	*VWA3B*	23	1.35	0.3	0.1	< 0.01	↑
P63261	*Actin, cytoplasmic 2*	*ACTG1*	456	1.32	0.28	0.06	< 0.01	↑
Q562R1	*Beta-actin-like protein 2*	*ACTBL2*	173	1.31	0.27	0.07	< 0.01	↑
P62736	*Actin, aortic smooth muscle*	*ACTA2*	238	1.31	0.27	0.07	< 0.01	↑
P60709	*Actin, cytoplasmic 1*	*ACTB*	456	1.30	0.26	0.06	< 0.01	↑
P0DTE7	*Alpha-amylase 1B*	*AMY1B*	4384	1.27	0.24	0.01	< 0.01	↑
P0DOX5	*Immunoglobulin gamma-1 heavy chain*	*IGG1*	122	1.26	0.23	0.15	0.02	↑
P19961	*Alpha-amylase 2B*	*AMY2B*	3418	1.26	0.23	0.01	< 0.01	↑
P04746	*Pancreatic alpha-amylase*	*AMY2A*	3027	1.26	0.23	0.01	< 0.01	↑
P0DUB6	*Alpha-amylase 1A*	*AMY1A*	4384	1.23	0.21	0.01	< 0.01	↑
P0DTE8	*Alpha-amylase 1C*	*AMY1C*	4384	1.23	0.21	0.01	< 0.01	↑
P63267	*Actin, gamma-enteric smooth muscle*	*ACTG2*	238	1.23	0.21	0.05	< 0.01	↑
Q6S8J3	*POTE ankyrin domain family member E*	*POTEE*	120	1.20	0.18	0.09	0.02	↑
P06702	*Protein S100-A9*	*S100A9*	236	1.17	0.16	0.06	< 0.01	↑
P01037	*Cystatin-SN*	*CST1*	5979	1.12	0.11	0.01	< 0.01	↑

Note: Log (e) (“e” is a constant = 2.71); SD, standard deviation; p, statistical significance (adjusted by False Discovery Rate - FDR=4); ED, Expression differences; ↑ = up-regulated in G1 (1-p>0.95). Bold lines indicate fold change higher than 2.

**Table 3:** Key downregulated proteins identified in G1 compared with G2.

Accession number	Protein name	Gene	Score	Fold Change	Log(e)	SD	p	ED
**P02808**	***Statherin***	***STATH***	**1181**	**5.15**	**-1.64**	**0.09**	**< 0.01**	**↓**
**P23280**	***Carbonic anhydrase 6***	***CA6***	**580**	**5.00**	**-1.61**	**0.05**	**< 0.01**	**↓**
**P05109**	***Protein S100-A8***	***S100A8***	**554**	**4.52**	**-1.51**	**0.03**	**< 0.01**	**↓**
**Q9UGM3**	***Deleted in malignant brain tumors 1 protein***	***DMBT1***	**31**	**3.15**	**-1.15**	**0.05**	**< 0.01**	**↓**
**P07737**	***Profilin-1***	***PFN1***	**491**	**2.94**	**-1.08**	**0.17**	**< 0.01**	**↓**
**P02814**	***Submaxillary gland androgen-regulated protein 3B***	***SMR3B***	**7816**	**2.69**	**-0.99**	**0.01**	**< 0.01**	**↓**
**Q96DA0**	***Zymogen granule protein 16 homolog B***	***ZG16B***	**3747**	**2.05**	**-0.72**	**0.03**	**< 0.01**	**↓**
**P04406**	***Glyceraldehyde-3-phosphate dehydrogenase***	***GAPDH***	**472**	**2.03**	**-0.71**	**0.07**	**< 0.01**	**↓**
**Q5VSP4**	***Putative lipocalin 1-like protein 1***	***LCN1P1***	**79**	**2.01**	**-0.7**	**0.06**	**< 0.01**	**↓**
P02810	*Salivary acidic proline-rich phosphoprotein 1/2*	*PRH1; PRH2*	3562	1.97	-0.68	0.03	< 0.01	↓
P59666	*Neutrophil defensin 3*	*DEFA3*	514	1.82	-0.6	0.1	0.03	↓
P06870	*Kallikrein-1*	*KLK1*	121	1.80	-0.59	0.09	< 0.01	↓
P06744	*Glucose-6-phosphate isomerase*	*GPI*	43	1.75	-0.56	0.11	< 0.01	↓
P31025	*Lipocalin-1*	*LCN1*	170	1.69	-0.53	0.06	< 0.01	↓
P13929	*Beta-enolase*	*ENO3*	44	1.63	-0.49	0.18	< 0.01	↓
P62937	*Peptidyl-prolyl cis-trans isomerase A*	*PPIA*	124	1.63	-0.49	0.16	< 0.01	↓
P54108	*Cysteine-rich secretory protein 3*	*CRISP3*	211	1.44	-0.37	0.12	0.02	↓
P01009	*Alpha-1-antitrypsin*	*SERPINA1*	92	1.33	-0.29	0.09	< 0.01	↓
P02647	*Apolipoprotein A-I*	*APOA1*	81	1.27	-0.24	0.08	0.01	↓
P01036	*Cystatin-S*	*CST4*	5005	1.20	-0.19	0.02	< 0.01	↓
P22079	*Lactoperoxidase*	*LPO*	67	1.18	-0.17	0.1	0.04	↓
B9A064	*Immunoglobulin lambda-like polypeptide 5*	*IGLL5*	1340	1.12	-0.12	0.06	0.04	↓
P61626	*Lysozyme C*	*LYZ*	1239	1.12	-0.12	0.07	0.04	↓
P0DOX8	*Immunoglobulin lambda-1 light chain*	*IGL1*	1340	1.12	-0.12	0.05	0.01	↓
P02768	*Albumin*	*ALB*	2966	1.11	-0.11	0.02	< 0.01	↓
P09228	*Cystatin-SA*	*CST2*	1072	1.08	-0.08	0.02	< 0.01	↓
P12273	*Prolactin-inducible protein*	*PIP*	4781	1.06	-0.06	0.03	0.01	↓

Note: Log (e) (“e” is a constant = 2.71); SD, standard deviation; p, statistical significance (adjusted by False Discovery Rate - FDR = 4); ED, Expression differences; ↓ = downregulated in G1 (p < 0.05). Bold lines indicate fold change higher than 2

Most up-regulated proteins in G1 were *Proline-rich protein 4*, which showed an 8-fold increase, followed by *Lactotransferrin*, *Cystatin-D*, and *Haptoglobin*, all exhibiting a 4-fold increase. Other notable up-regulated proteins included *Basic salivary proline-rich proteins 1* and *2*, *Beta-2-microglobulin*, *Serotransferrin*, and *Zinc-alpha-2-glycoprotein*, which were increased 2-fold. Additionally, several immunoglobulin isoforms showed significant up-regulation, including *Immunoglobulin heavy constant gamma 4* (5-fold increase), *Immunoglobulin heavy constant gamma 2* (4-fold increase), *Immunoglobulin mu heavy chain*, *Immunoglobulin lambda constant 7*, *Immunoglobulin J chain* (3-fold increase), as well as *Immunoglobulin kappa light chain*, *Immunoglobulin kappa constant*, and *Immunoglobulin lambda constant 6*, which showed a 2-fold increase (Table 2).

*Statherin* and *Carbonic anhydrase 6*, both showing a 5-fold decrease, were the most down regulated proteins in G1. *Protein S100-A8* exhibited a 4-fold reduction, whereas the *Deleted in malignant brain tumors 1* protein decreased 3-fold. Additional proteins with notable downregulation included *Profilin-1*, *Submaxillary gland androgen-regulated protein 3B*, *Zymogen granule protein 16 homolog B*, *Glyceraldehyde-3-phosphate dehydrogenase*, and *Putative lipocalin 1-like protein 1*, all showing a 2-fold decrease (Table 3).

**Figure 2:**
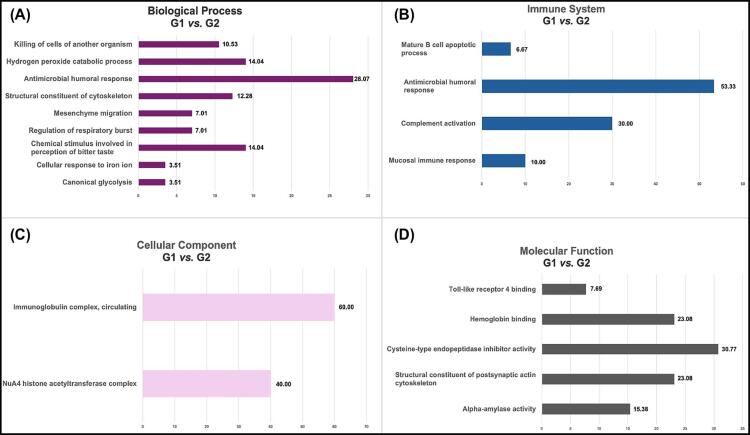
Functional analysis comparing G1 and G2. Differential expression patterns varied across categories, with both up-regulated and downregulated proteins in G1 compared with G2. (A) Biological Processes highlighting the antimicrobial humoral response and the hydrogen peroxide catabolic process. (B) Immune System Processes emphasizing the antimicrobial humoral response and complement activation. (C) Cellular Component analysis showing predominant categories like the immunoglobulin complex, circulating and the NuA4 histone acetyltransferase complex. (D) Molecular Function analysis highlighting cysteine-type endopeptidase inhibitor activity.

Figure 2 presents the functional analysis comparing G1 and G2. We identified biological processes related to the antimicrobial humoral response (28.07%; $p=7.5\times 10^{-17}$ ) and the hydrogen peroxide catabolic process (14.04%; $p=8.1\times 10^{-11}$ ). Regarding the immune system, notable categories included the antimicrobial humoral response (53.33%; $p=5.2\times 10^{-12}$ ) and complement activation (30%; $p=2.2\times 10^{-7}$ ). For cellular component the predominant categories were the immunoglobulin complex, circulating (60%; $p=7.5\times 10^{-19}$ ) and the NuA4 histone acetyltransferase complex (40%; $p=1.8\times 10^{-8}$ ). Regarding molecular function, the most significant category was cysteine-type endopeptidase inhibitor activity (30.77%; $p=3.1\times 10^{-11}$ ) (Figure 2).

**Figure 3:**
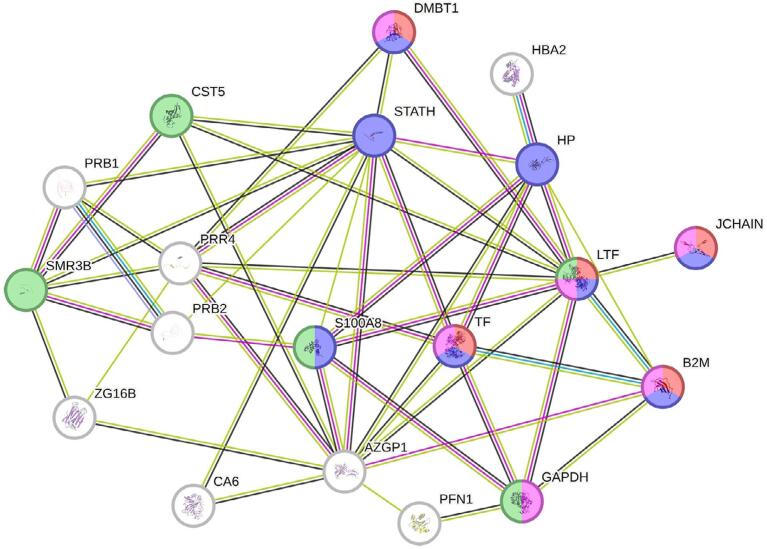
Interaction network of up- and downregulated proteins in G1 compared with G2 (changes >2-fold), highlighting functional categories and associated proteins. Blue nodes represent proteins linked to the defense response to bacteria; red and pink nodes indicate proteins involved in antibacterial and antimicrobial humoral responses, respectively; and green nodes highlight proteins associated with endopeptidase regulation. Acronyms shown in the figure correspond to gene symbols for each protein, which are detailed in Tables 2 and 3.

Figure 3 illustrates the interaction network of up- and downregulated proteins (with changes greater than 2-fold) in G1 compared with G2. Blue nodes highlight proteins linked to the defense response to bacterium ( $FDR=1.63\times 10^{-6}$ ), such as *Lactotransferrin* (increased 5-fold), *Haptoglobin* (increased 4-fold), *Immunoglobulin J chain* (increased 3-fold), *Serotransferrin* (increased 2-fold), *Beta-2-microglobulin* (increased 2-fold), *Statherin* (decreased 5-fold), *Protein S100-A8* (decreased 4-fold), and *Deleted in malignant brain tumors 1 protein* (decreased 3-fold). Red and pink nodes represent proteins related to the antibacterial and antimicrobial humoral responses ( $FDR=1.76\times 10^{-5}$ ), respectively, including *Lactotransferrin*, *Immunoglobulin J chain*, *Beta-2-microglobulin*, *Serotransferrin*, *Deleted in malignant brain tumors 1 protein*, and *Glyceraldehyde-3-phosphate dehydrogenase* (decreased 2-fold). Lastly, green nodes emphasize proteins associated with endopeptidase regulation (*FDR*=0.0304), including *Lactotransferrin*, *Cystatin-D* (increased 4-fold), *Protein S100-A8*, *Glyceraldehyde-3-phosphate dehydrogenase*, and *Submaxillary gland androgen-regulated protein 3B* (decreased 2-fold).

## Discussion

We explored the salivary proteomic profile associated with generalized gingivitis in pregnant women with obesity, shedding light on the biological mechanisms underlying this condition. A notable strength of this study was the rigorous recruitment criteria, ensuring highly homogeneous groups regarding age, socioeconomic status, anthropometric parameters, oral hygiene behaviors, and periodontal clinical measures. Despite this homogeneity, we identified significant salivary proteomic differences in obese pregnant women with generalized gingivitis, reinforcing the potential of salivary proteomics as a non-invasive approach for identifying gingival inflammation biomarkers in high-risk populations. Proteins like *Proline-rich protein 4*, *Lactotransferrin*, *Serotransferrin*, *Haptoglobin*, *Statherin*, and *Protein S100-A8* emerge as promising candidates for future diagnostic panels in this population, paving the way for improved clinical management and personalized care.

To ensure the robustness of these findings, our proteomic analysis relied on PLGS software which applies Monte Carlo algorithms and Bayesian probability adjustments to estimate differential protein expression. This approach enhances data reliability by iteratively refining the probable presence of each protein based on observed spectra, minimizing false positives and optimizing the identification of biologically relevant proteins. Such methodological strategy strengthens the interpretation of our findings, ensuring that the identified protein alterations truly reflect biological differences rather than analytical artifacts.

Functional analysis revealed that differential protein expressed in pregnant women with obesity and generalized gingival inflammation are primarily involved in antimicrobial humoral responses and hydrogen peroxide catabolism, processes critical for host defense and oxidative stress regulation. Generalized gingivitis was associated with higher expression of *Proline-rich proteins 1*, *2*, and *4*, with *Proline-rich protein 4* showing an over 8-fold increase in G1. *Proline-rich proteins* (PRPs), predominantly produced by the parotid and submandibular glands, constitute a significant portion of total salivary proteins. These proteins play a dual role in bacterial agglutination, contributing to biofilm regulation.^23^*Proline-rich protein 4*, typically associated with lacrimal glands but also expressed in other exocrine tissues, highlights overlapping roles in host defense.^24^^,^^25^ These findings suggest that PRPs, particularly *Proline-rich protein 4*, may protect against bacterial adhesion and reflect an active defense mechanism against biofilm formation.

The primary immune pathways enriched in this study were ‘antimicrobial humoral response’ and ‘complement activation.’ The former refers to immune mechanisms mediated by soluble molecules, with 53.33% of genes from these differentially expressed proteins directly involved in this function mediated by antimicrobial peptides, immunoglobulins, and proteins that neutralize, agglutinate, or eliminate pathogenic microorganisms. High salivary expression of 15 immunoglobulin isoforms in G1, with some showing over a 5-fold increase (e.g., *Immunoglobulin heavy constant gamma 4*), reflects an amplified immune response likely targeting biofilm-associated antigens. Our findings indicate that the heightened humoral immune response and complement activation observed in G1, by up-regulation of immunoglobulins and antimicrobial proteins, reflects an intensified inflammatory response which may contribute to vascular changes and increased permeability of gingival tissues, ultimately leading to bleeding.^13^^,^^26^

‘Complement activation’, integral to innate immunity, was enriched in nearly one-third of the genes from differentially expressed proteins, highlighting its pivotal role in the observed immune response. This pathway eliminates pathogens via three main mechanisms: opsonization, marking them for phagocytosis by immune cells; formation of the membrane attack complex, leading to pathogen cell lysis; and recruitment of immune cells to the infection site.^27^ Elevated expression of *Serotransferrin*, *Beta-2-microglobulin*, *Haptoglobin*, and *Lactotransferrin* in G1 underline their roles in bacterial defense and inflammation. Notably, *Lactotransferrin* inhibits bacterial growth by binding iron and limiting its availability.^28^ Its nearly 5-fold increase in G1 reinforces its importance in immune responses linked to generalized gingival inflammation. Recent findings align with our results showing a 1.6-fold increase in *Lactotransferrin* in individuals with gingivitis, reflecting its role in countering bacterial biofilm formation.^29^

*Serotransferrin*, in turn, is a key marker of blood contamination in saliva.^30^ Its increased presence in whole-mouth saliva suggests greater vascular permeability and inflammatory exudation in compromised gingival tissues, supporting its role as a potential biomarker of gingival bleeding severity.^30^*Beta-2-microglobulin* may be an important salivary marker of compromised mucosal integrity in gingivitis, although some aspects of this association remain unclear. Produced and released by various cells, especially lymphocytes, its presence in saliva reflects increased cell membrane turnover.^31^^-^^33^ These markers linked to inflammatory and exudative processes in periodontal tissues showed over a 2-fold increase in G1, highlighting their potential as biomarkers for gingival inflammation and tissue damage.

*Haptoglobin* was another up-regulated protein with nearly 4-fold increase in pregnant women with obesity and generalized gingivitis. It may play an important role in controlling inflammation and aiding tissue recovery in response to gingival challenges, although its role in gingival bleeding is poorly understood. *Haptoglobin* has antioxidant and antibacterial properties, binds free hemoglobin, and helps macrophages clear it, providing bacteriostatic and antioxidant effects.^34^ It also supports tissue repair during infections by reducing inflammation which may be vital in the periodontal environment.^35^

Conversely, the downregulation of *Statherin* and *Carbonic Anhydrase 6* (5-fold decrease) in G1 suggests disrupted oral homeostasis. *Statherin* plays a crucial role in biofilm regulation whereas *Carbonic Anhydrase 6* is essential for pH balance, emphasizing their combined importance in mitigating oral inflammation. *Statherin* specifically inhibits the growth of anaerobic bacteria, such as *Porphyromonas gingivalis* and *Fusobacterium nucleatum*, and facilitates selective bacterial colonization on supragingival and subgingival tooth surfaces.^36^ Reduced *Statherin* levels in G1 may compromise the antibacterial defenses of saliva, potentially contributing to the onset of gingival inflammation as recently highlighted by Parlak, et al.^36^ (2023). Further studies are necessary to confirm *Statherin* as a salivary biomarker for severe gingival inflammation, particularly in pregnant women with obesity.

The 4-fold decrease in *Protein S100-A8*, a key inflammation modulator, suggests a dysregulated immune response in G1. This unexpected downregulation observed in pregnant women with obesity and generalized gingivitis could indicate suppressed immune activation or an insufficient response to bacterial challenges, as previously reported.^37^*S100A8* primarily acts extracellularly as a heterodimer with *S100A9*, forming *calprotectin* which is crucial for immune activation, apoptosis, and inflammation regulation in periodontal tissues. Its reduced expression may also reflect altered *calprotectin* activity, signaling an adaptive shift or impairment in the inflammatory response during pregnancy-associated gingival inflammation. These findings highlight the need for further investigation into the roles of *S100A8* and *calprotectin* in this unique immunological context.^37^

Functional analysis revealed significant enrichment of the immunoglobulin complex as a cellular component and increased activity of cysteine-type endopeptidase inhibitors. These findings highlight the critical interplay between immune modulation and protease inhibition in controlling inflammation and maintaining tissue integrity. Proteins like *Lactotransferrin* and *Cystatin-D*, which showed a 4-fold increase, contrast with the downregulation of *S100-A8*, *Glyceraldehyde-3-phosphate dehydrogenase*, and *Submaxillary gland androgen-regulated protein 3B*. This dynamic balance between protease regulation and immune response underlines the complexity of gingival inflammation during pregnancy, as these shifts in protein expression may play a crucial role in periodontal homeostasis, particularly in high-risk populations.

Pregnant women with obesity and generalized gingival bleeding exhibited notably higher expression of *Cystatin-D* (>4-fold), with slight increases in *Cystatin-B* (1.62-fold), *Cystatin-C* (1.49-fold), and *Cystatin-SN* (1.12-fold) compared with G2. However, G1 showed slightly lower expression of *Cystatin-SA* (1.08-fold lower) and *Cystatin-S* (1.20-fold lower). These findings partly contrast with those of Balan, et al.^13^ (2022), who reported reduced levels of *Cystatins* (*S*, *SA*, and *SN*) in pregnant women with gingivitis, alongside decreases in *Cystatin-C* and *Cystatin-D*. Notably, Balan, et al.^13^ (2022) identified *Cystatin-C* as a critical regulator in major catabolic pathways and a key pregnancy gingivitis modulator.

Discrepancies between our findings and those of Balan, et al.^13^ (2022) suggest that cystatins may play dual roles in periodontal health, with some acting protectively by inhibiting protease activity and others reflecting adaptive responses to inflammation. In our study, the pronounced up-regulation of *Cystatin-D* in G1 may represent a compensatory mechanism to mitigate inflammation, whereas the slight downregulation of *Cystatin-S* and *Cystatin-SA* suggests compromised biofilm regulation. But despite statistical significance for all intergroup comparisons, only *Cystatin-D* showed a fold change above two. These findings should therefore be interpreted with caution. Nonetheless, our results emphasize the potential of cystatins as biomarkers for periodontal inflammation and highlight the need for further research to elucidate their roles in pregnancy-associated gingival inflammation.

This study presents some limitations. First, generalized gingival bleeding, as assessed in this study, should not be interpreted as a definitive clinical diagnostic criterion. Rather, by considering only cases with BOP ≥ 50% and excluding the 30–50% range we focused on a more severe form of gingivitis, enabling the identification of differentially expressed proteins and the biological mechanisms associated with a more severe gingival inflammatory pattern. Although our study included a control group composed of obese pregnant women without generalized gingivitis, we did not include a group of eutrophic pregnant women (with or without generalized gingivitis). Future studies should investigate these outcomes in eutrophic individuals to enable broader comparisons and better contextualize our findings.

Second, since unstimulated whole saliva was collected instead of saliva directly from the gland’s duct, some contribution of gingival crevicular fluid (particularly in cases of inflammation) cannot be ruled out. The use of unstimulated saliva was based on a previous study conducted by our group,^21^ in which we compared the proteomic profiles of stimulated and unstimulated saliva in obese and eutrophic pregnant women (with and without periodontitis). That study showed that saliva stimulation decreased important proteins involved in immune response and inflammation across all groups, pointing to unstimulated saliva as the best choice for proteomic analysis in pregnant women.^21^ However, we acknowledge that this comparison was not performed specifically in the present sample of obese pregnant women with and without generalized gingivitis. Future studies should address this gap to confirm whether unstimulated saliva is the most suitable option in this context. A third limitation is the study’s cross-sectional design which does not allow for causal inferences. However, despite the sample’s homogeneity, its small size may limit generalizing the findings to a broader population. Future studies should consider longitudinal designs to track proteomic changes throughout pregnancy, explore the efficacy of targeted interventions, and integrate microbiome analyses to provide a more comprehensive understanding of the host-microbiome interplay in this population.

Despite these limitations, our findings have important clinical implications as they highlight the potential of salivary biomarkers, like *Proline-rich protein 4*, *Lactotransferrin*, and *Serotransferrin*, as non-invasive tools for detecting and monitoring severe gingival inflammation in high-risk pregnant women. Given the association between immune dysregulation, oxidative stress, and increased gingival permeability, these biomarkers could aid in identifying patients who may benefit from early periodontal interventions, potentially reducing adverse pregnancy outcomes. Inclusion of additional candidates such as *Haptoglobin*, *Statherin*, and *Protein S100-A8*, may further refine periodontal risk assessment during prenatal care. Integrating salivary diagnostics into routine maternal healthcare could enhance early identification of periodontal complications, supporting personalized preventive and therapeutic strategies. Future studies should validate these biomarkers in clinical settings to ensure their applicability in obstetric and dental care.

## Conclusion

Pregnant women with obesity and generalized gingivitis exhibited distinct salivary proteomic profiles marked by up-regulation of immune-related proteins and downregulation of tissue-protective proteins. These findings underline the potential of salivary proteomics for identifying periodontal inflammation biomarkers, paving the way for detection and personalized management strategies in this high-risk group. Future studies are needed to validate these biomarkers and explore targeted interventions to mitigate periodontal risks during pregnancy.
